# Development and external validation of a novel nomogram for predicting cancer-specific survival in patients with ascending colon adenocarcinoma after surgery: a population-based study

**DOI:** 10.1186/s12957-022-02576-4

**Published:** 2022-04-19

**Authors:** Yi Fan Zhang, Cheng Ma, Xiao Ping Qian

**Affiliations:** 1 Comprehensive Cancer Center, Nanjing Drum Tower Hospital Clinical College of Nanjing Medical University, Nanjing, 210000 China; 2 Department of Radiotherapy, The Xuzhou School of Clinical Medicine of Nanjing Medical University, Xuzhou, 221000 China; 3 Department of Gastrointestinal Surgery, The Xuzhou School of Clinical Medicine of Nanjing Medical University, Xuzhou, 221000 China; 4 Comprehensive Cancer Center, Nanjing Drum Tower Hospital, Medical School of Nanjing University, Clinical Cancer Institute of Nanjing University, Nanjing, 210000 China

**Keywords:** Nomogram, Cancer-specific survival (CSS), Ascending colon adenocarcinoma, Predict, Lymph node ratio (LNR)

## Abstract

**Background:**

This study aimed to develop and validate a novel nomogram to predict the cancer-specific survival (CSS) of patients with ascending colon adenocarcinoma after surgery.

**Methods:**

Patients with ascending colon adenocarcinoma were enrolled from the Surveillance, Epidemiology, and End Results (SEER) database from 1973 to 2015 and randomly divided into a training set (5930) and a validation set (2540). The cut-off values for age, tumour size and lymph node ratio (LNR) were calculated via X-tile software. In the training set, independent prognostic factors were identified using univariate and multivariate Cox analyses, and a nomogram incorporating these factors was subsequently built. Data from the validation set were used to assess the reliability and accuracy of the nomogram and then compared with the 8th edition of the American Joint Committee on Cancer (AJCC) tumour-node-metastasis (TNM) staging system. Furthermore, external validation was performed from a single institution in China.

**Results:**

A total of 8470 patients were enrolled from the SEER database, 5930 patients were allocated to the training set, 2540 were allocated to the internal validation set and a separate set of 473 patients was allocated to the external validation set. The optimal cut-off values of age, tumour size and lymph node ratio were 73 and 85, 33 and 75 and 4.9 and 32.8, respectively. Univariate and multivariate Cox multivariate regression revealed that age, AJCC 8th edition T, N and M stage, carcinoembryonic antigen (CEA), tumour differentiation, chemotherapy, perineural invasion and LNR were independent risk factors for patient CSS. The nomogram showed good predictive ability, as indicated by discriminative ability and calibration, with *C* statistics of 0.835 (95% CI, 0.823–0.847) and 0.848 (95% CI, 0.830–0.866) in the training and validation sets and 0.732 (95% CI, 0.664–0.799) in the external validation set. The nomogram showed favourable discrimination and calibration abilities and performed better than the AJCC TNM staging system.

**Conclusions:**

A novel validated nomogram could effectively predict patients with ascending colon adenocarcinoma after surgery, and this predictive power may guide clinicians in accurate prognostic judgement.

## Introduction

Colorectal cancer (CRC) is the third most commonly diagnosed cancer and the second leading cause of cancer-related death worldwide, with the predominant type being adenocarcinoma [1]. In the USA, there were an estimated 104,270 new cases and 52,980 deaths due to colon cancer in 2021 [2]. The colon can be divided into the left and right colon according to the localization of the primary tumour, with the splenic flexure as the boundary. The right side of the colon is historically derived from the embryological midgut, and the left side originates in the embryological hindgut. Epidemiological, clinical and molecular biological differences between left-sided and right-sided colon cancer have been elucidated and studied in many studies [3, 4]. It has been suggested that left and right-sided colon cancer may be two different entities [5]. Right-sided colon tumours usually exhibit larger size and higher tumour grade, leading to worse prognosis compared to left-sided cancer [5, 6]. The caecum and ascending colon are considered the most common tumour sites in right-sided colon [7]. Unlike the caecum, which develops from the caecal diverticulum in the 5-week-old embryo, the ascending colon arises from the caudal limb of the midgut loop. There may be differences between caecum and ascending colon carcinomas due to different locations and developmental processes. The colonic subsite should be taken into consideration when cancer is classified [7]. Further detailed analysis may be necessary to investigate the epidemiology and prognosis of tumours in different areas of the colon to facilitate a more precise and individualized treatment plan and the prediction of survival.

Surgical resection and adjuvant chemotherapy are the mainstay treatments for patients with colon cancer. The pathological data and lymph node status guide prognosis and adjuvant therapy recommendations [8], defined as the proportion of positive regional nodes among the number of examined regional nodes, and have been demonstrated to have a significant prognostic role in many cancers [9–12], even superior to pN stage in many studies [13].

The American Joint Committee on Cancer (AJCC) tumour-node-metastasis (TNM) staging system is recognized and widely used in predicting the prognosis of tumours. However, there are limitations concerning TNM classification because all colorectal tumours share the same stage standards even when they do not fit into either subdivision. The TNM staging system cannot serve as the best prognostic tool since it only includes the anatomical information of the tumour and ignores the clinical features and laboratory tests of the patients.

A nomogram is a graphical predictive tool with a calculated score that is used to predict tumour prognosis, and it can provide individualized, evidence-based, accurate risk estimation [14]. Furthermore, nomograms improve the decision-making processes and are relatively easy to use. A number of nomograms have been developed to predict outcomes in various types of malignancies. Nevertheless, no data were available in nomograms including LNR to predict the prognosis in ascending colon adenocarcinoma after surgery. Cancer-specific survival (CSS), which directly reflects cancer prognosis, is calculated from diagnosis to death from the tumour. In this study, we aimed to assess the predictive ability of a nomogram incorporating the LNR in patients with ascending colon adenocarcinoma after surgery. In addition, we also compared the predictive accuracy and discriminability of the nomogram for predicting CSS with the current TNM staging system.

## Materials and methods

### Patients

Data from patients with ascending colon adenocarcinoma were extracted from the SEER database using SEER*stat software (version 8.3.6, NCI, Bethesda, USA). The SEER database collects patient demographics and publishes cancer incidence and survival data, covering approximately 34% of the US population. Patient data was collected for those diagnosed with ascending colon adenocarcinoma or included an adenocarcinoma as a component of their primary malignancy (C18.2) between 2004 and 2015, and histological classification was based on the International Classification of Diseases Codes for Oncology (ICD-O) proposed in 2000 (8140/3,8144/3,8210/3,8211/3,8213/3,8220/3,8244/3,8255/3,8260/3,8261/3,8262/3,8263/3,8323/3,8480/3,8481/3,8560/3,8574/3). The inclusion criteria were as follows: only one primary tumour after surgery; definite age, race, tumour size and pathology data; complete information of AJCC TNM stage, follow-up, CSS time and status; and detailed information about regional nodes, level of CEA and perineural invasion. An external validation set complying with the above criteria was collected from The Central Hospital of Xuzhou, Affiliated Hospital of Nanjing Medical University (from 2011 to 2020). Patients were reclassified according to the 8th edition of the TNM classification based on the 7th edition data provided by the SEER database. All methods were performed in accordance with the relevant guidelines and regulations of the SEER database. Written informed consent for publication was obtained from all participants, and ethics committee approval was obtained from a local ethics committee.

### Statistical analysis

Statistical analysis was carried out using SPSS statistics software, version 25.0 (SPSS, Chicago, IL, USA), and R version 3.6.2 software (The R Foundation for Statistical Computing, Vienna, Austria.http://www.r-project.org). The optimum cut-off values for LNR, age and tumour size were generated by X-tile version 3.6.1 software (Yale University School of Medicine, New Haven, Conn). Variables with a *p* value <0.05 in the univariate analysis were entered into the multivariate analysis via the Cox proportional hazards model in the training set. A nomogram for 1-, 3- and 5-year CSS was constructed according to the results of multivariate survival analysis. The discrimination accuracy was measured by Harrell’s concordance index (C-index) and receiver operating characteristic (ROC) curves, and the corresponding areas under the curves (AUCs) were computed. Time-dependent ROC analysis was also performed. Calibration curves were generated to assess the consistency of the nomogram by using the bootstrap method (resampling = 1000) in the training and validation sets. Decision curve analysis (DCA) [15], a novel method to evaluate prediction models by calculating the clinical net benefit, was conducted for decision making in both the internal and external validation cohorts. The performance of the nomogram was compared with the traditional AJCC 8th TNM staging system. *P* values <0.05 were considered statistically significant.

## Results

### Clinicopathologic and follow-up data

**Fig. 1:**
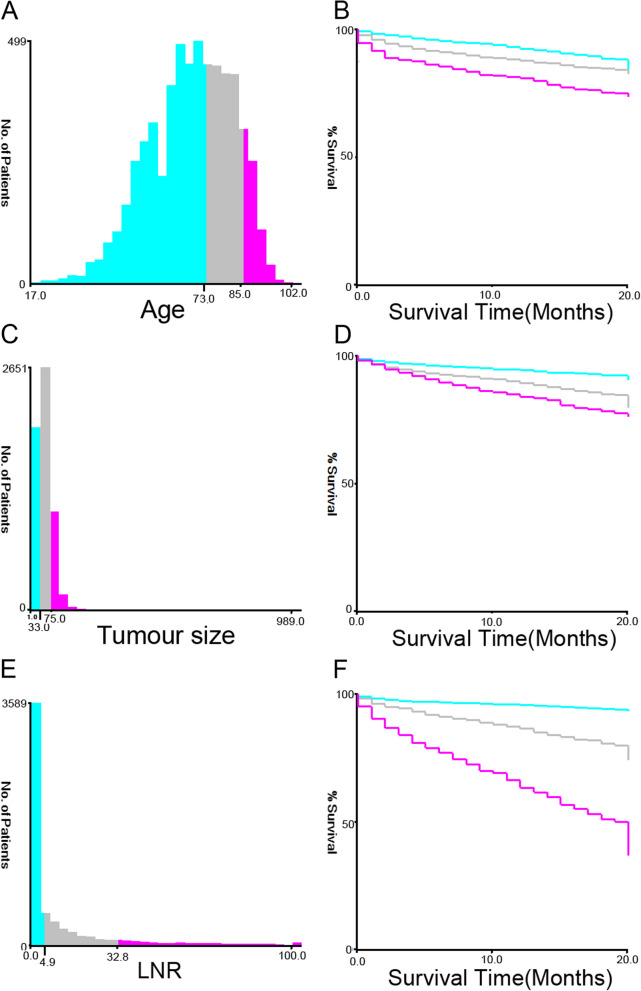
The X-tile analysis in the training cohort. The histograms showed the optimal cut-off point of age (**A**), tumour size (**C**) and LNR (**E**), and corresponding Kaplan-Meier analysis curves (**B**, **D**, **F**) were developed based on these cut-off points

A total of 8470 patients from the SEER database and 473 patients from a single centre in China were eventually enrolled in this study. Patients from SEER were randomly divided into a training set (*n*=5930) and an internal validation set (*n*=2540) at a ratio of 7t3. The mean age of the patients was 68.6 years, with males accounting for 45.7%. In terms of race, 6575 (77.6%) patients were white, 1170 (13.8%) were black and 725 (8.5%) were of other races. A minority of the patients exhibited increased CEA levels (3447, 40.6%) and perineural invasion (965, 14.7%). Of all 8470 patients, 594 (7.0%) were well differentiated, 5842 (69.0%) were moderately differentiated, 1681 (19.8%) were poorly differentiated and 353 (4.2%) were undifferentiated. The distribution of the 8th TNM stage among the patients was as follows: stage 0 and I, 1605 (18.9%); stage II, 3077 (36.3%); stage III, 2645 (31.2%); and stage IV, 1143 (13.5%). Postoperative pathological tumour staging suggested that 719 (8.4%) patients in the Tis and T1 stage, 1188 (14.0%) patients in the T2 stage, 5213 (61.5%) in the T3 stage and 1350 (15.9%) were in the T4 stage, while 4825 (57.0%) patients were in the N0 stage, 2172 (25.6%) in the N1 stage and 1473 (17.4%) in the N2 stage. In total, 1143 patients (13.4%) showed metastasis on pathology, and 5555 (65.5%) received adjuvant chemotherapy. According to the X-tile programme from the training set, the tumour size distribution was classified as ≤3.3 cm, 3.3–7.5 cm and >7.5 cm; the age distribution was classified as ≤73, 73–85 and >85; and the LNR distribution was classified as ≤4.9%, 4.9–32.8% and >32.8% (Fig. 1). Patient demographics and clinical and pathological characteristics of the training, internal and external validation sets are presented in Table 1.

**Table 1:** Baseline characteristics of the training, internal validation and external validation sets

Variables	Training set	Internal validation set	External validation set
	*n* = 5930 (%)	*n* = 2540 (%)	*n* = 473 (%)
Age			
≤73	3593 (60.6)	1585 (62.4)	345 (72.9)
73–85	1713 (28.9)	723 (28.5)	110 (23.3)
>85	624 (10.5)	232 (9.1)	18 (3.8)
Race			
White	4582 (77.3)	1993 (78.5)	0 (0.0)
Black	840 (14.2)	330 (13.0)	0 (0.0)
Other	508 (8.6)	217 (8.5)	473 (100.0)
Gender			
Male	2683 (45.2)	1192 (46.9)	255 (53.9)
Female	3247 (54.8)	1348 (53.1)	218 (46.1)
Differentiation			
Well	410 (6.9)	184 (7.2)	5 (1.1)
Moderate	4083 (68.9)	1759 (69.3)	432 (91.3)
Poor	1199 (20.2)	482 (19.0)	36 (7.6)
Undifferentiation	238 (4.0)	115 (4.5)	0 (0.0)
Stage_T			
T0/1	504 (8.5)	215 (8.5)	6 (1.3)
T2	847 (14.3)	341 (13.4)	91 (19.2)
T3	3654 (61.6)	1559 (61.4)	286 (60.5)
T4	925 (15.6)	425 (16.7)	90 (19.0)
Stage_N			
N0	3389 (57.2)	1436 (56.5)	308 (65.1)
N1	1520 (25.6)	652 (25.7)	118 (25.0)
N2	1021 (17.2)	452 (17.8)	47 (9.9)
Stage_M			
M0	5128 (86.5)	2199 (86.6)	439 (92.8)
M1	802 (13.5)	341 (13.4)	34 (7.2)
AJCC-TNM classification			
0/I	1140 (19.2)	465 (18.3)	69 (14.6)
II	2145 (36.2)	932 (36.7)	
III	1843 (31.1)	802 (31.6)	132 (27.9)
IV	802 (13.5)	341 (13.4)	34 (7.2)
CEA			
Normal	3516 (59.3)	1507 (59.3)	270 (57.1)
Elevate	2414 (40.7)	1033 (40.7)	203 (42.9)
Chemotherapy			
Yes	2021 (34.1)	894 (35.2)	116 (24.5)
No	3909 (65.9)	1646 (64.8)	357 (75.5)
Tumour size (cm)			
≤3.3	1644 (27.7)	680 (26.8)	110 (23.2)
3.3–7.5	3421 (57.7)	1489 (58.6)	319 (67.4)
>7.5	865 (14.6)	371 (14.6)	44 (9.3)
LNR			
≤4.9%	3785 (63.8)	1624 (63.9)	325 (68.7)
4.9–32.8%	1523 (25.7)	666 (26.2)	96 (20.3)
>32.8%	622 (10.5)	250 (9.8)	52 (11.0)
Perineural invasion			
No	5275 (89.0)	2230 (87.8)	461 (97.5)
Yes	655 (11.1)	310 (12.2)	12 (2.5)

CSS was defined as the survival time from diagnosis to cancer-associated death (CSD) and death from other causes, or those still alive were censored on the date of last follow-up. Based on the follow-up data, CSD was observed in 1745 patients from SEER and 64 in external validation (Fig. 1).

### Nomogram construction and validation

**Fig. 2:**
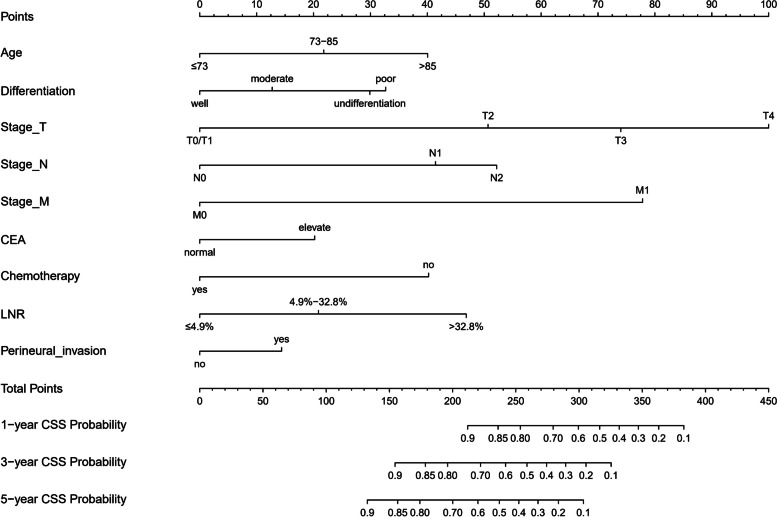
Nomogram for prediction of 1-, 3- and 5-year CSS rates of patients with ascending colon adenocarcinoma after surgery

**Fig. 3:**
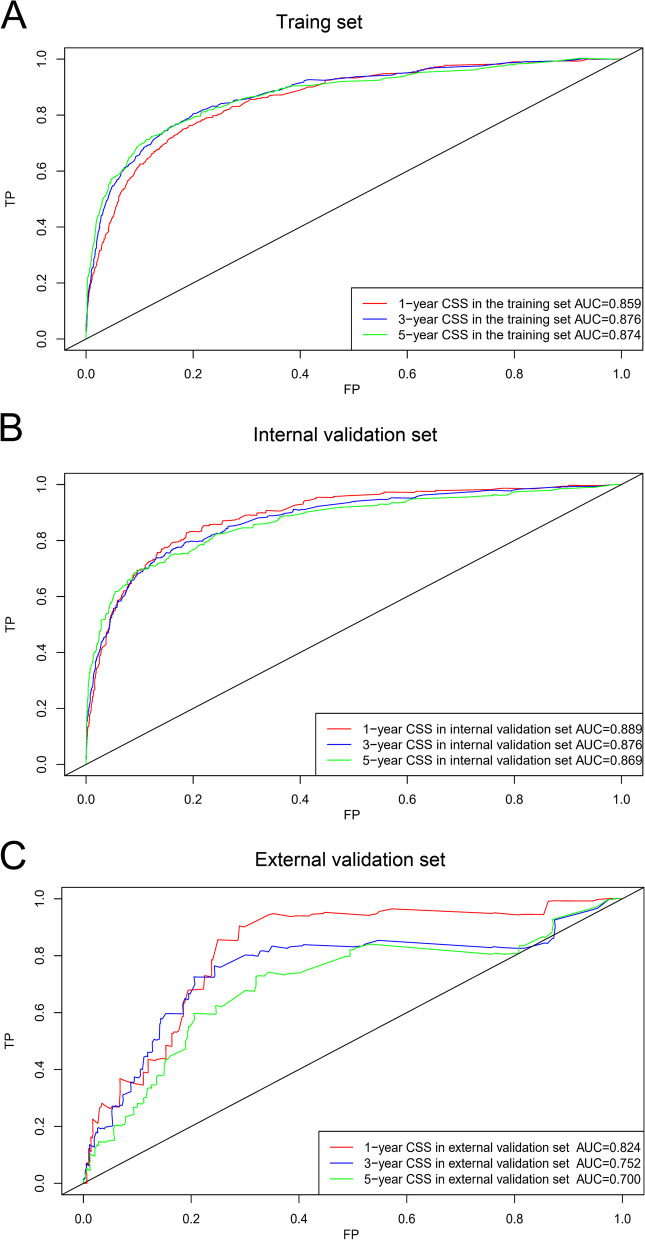
ROC curves of 1-, 3- and 5-year survival for predicting CSS in the training (**A**), internal validation (**B**) and external validation (**C**) sets

**Fig. 4:**
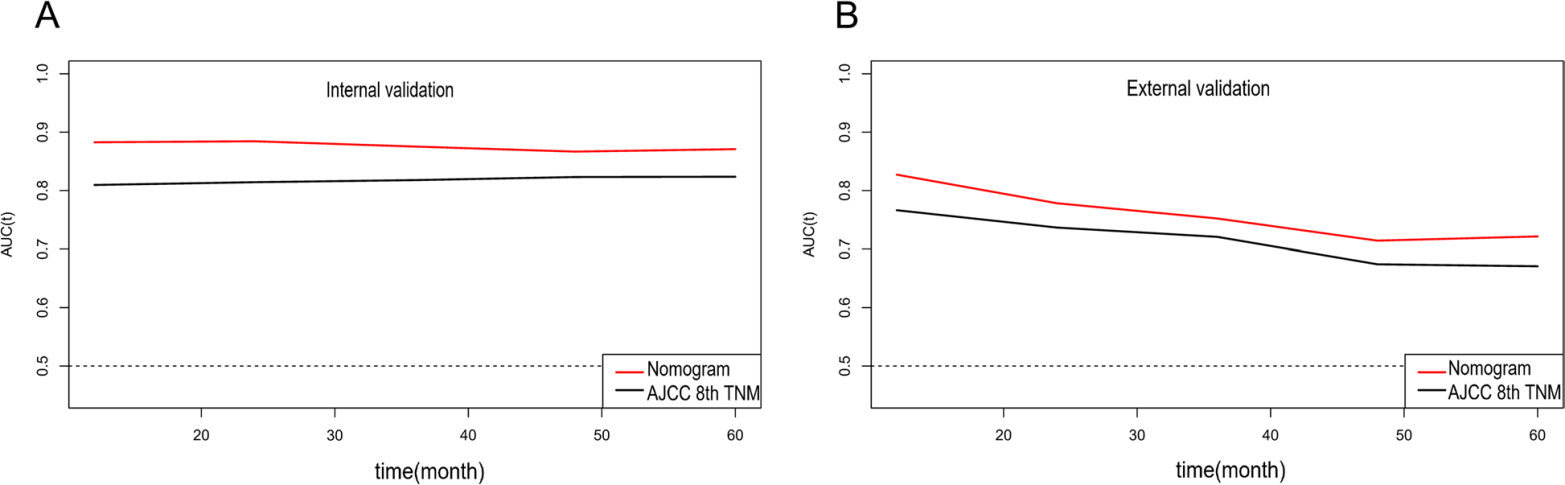
Time-dependent ROC curves of the nomogram and AJCC-TNM system in internal (**A**) and external (**B**) validation sets. Compared with the AJCC TNM 8th edition staging system, both curves of the nomogram were higher, showing a high predictive power

Univariate and multivariate Cox proportional hazard analyses revealed that age, serum CEA level, AJCC 8th edition T stage, N stage, M stage, chemotherapy, tumour differentiation and LNR were independent predictors of CSS (Table 2). The corresponding nomogram was constructed on the basis of the results of Cox regression analyses (Fig. 2). The C-index of the nomogram for CSS prediction was 0.835 (95% CI, 0.823–0.847), 0.848 (95% CI, 0.830–0.866) and 0.732 (95% CI, 0.664–0.799) in the training, internal validation and external validation sets, respectively. The C-index of the AJCC TNM 8th edition staging system was 0.787 (95% CI, 0.767–0.807) and 0.701 (95% CI, 0.648–0.754) in the internal and external validation sets, respectively. The AUCs of 1-, 3- and 5-year CSS prediction were 0.859, 0.876 and 0.874 in the training set; 0.889, 0.876 and 0.869 in the internal validation set; and 0.824, 0.752 and 0.700 in the external validation set, respectively (Fig. 3). Furthermore, the two time-dependent ROC curves of the nomogram were higher than those of the TNM staging system (Fig. 4), indicating comparative stability and adequate discriminability in both the internal and external validation sets.

**Table 2:** Univariate and multivariate analysis of parameters related with CSS in training set

Variables	Univariate analysis			Multivariate analysis		
	HR	95%Cl	*p*	HR	95%Cl	*p*
Age						
≤73	1					
73–85	**1.511**	**1.320–1.731**	**<0.001**	**1.482**	**1.295–1.695**	**<0.001**
>85	**2.135**	**1.783–2.558**	**<0.001**	**2.06**	**1.724–2.462**	**<0.001**
Race						
White	1					
Black	1.16	0.990–1.360	0.067			
Other	1.037	0.851–1.264	0.718			
Gender (male/female)	0.941	0.838–1.056	0.301			
Differentiation						
Well	1					
Moderate	1.264	0.913–1.748	0.158	1.258	0.910–1.741	0.165
Poor	**1.807**	**1.295–2.521**	**<0.001**	**1.804**	**1.292–2.517**	**<0.001**
Undifferentiation	**1.771**	**1.205–2.604**	**0.004**	**1.715**	**1.168–2.520**	**0.006**
Stage_T						
T0/1	1					
T2	**2.632**	**1.418–4.887**	**0.002**	**2.519**	**1.360–4.665**	**0.003**
T3	**3.966**	**2.204–7.138**	**<0.001**	**3.837**	**2.150–6.848**	**<0.001**
T4	**6.291**	**3.459–11.440**	**<0.001**	**6.134**	**3.404–11.055**	**<0.001**
Stage_N						
N0	1					
N1	**2.129**	**1.625–2.790**	**<0.001**	**2.112**	**1.612–2.768**	**<0.001**
N2	**2.622**	**1.892–3.633**	**<0.001**	**2.565**	**1.851–3.554**	**<0.001**
Stage_M(M0/M1)	**4.157**	**3.611–4.784**	**<0.001**	**4.074**	**3.541–4.686**	**<0.001**
CEA (negative/positive)	**1.417**	**1.250–1.607**	**<0.001**	**1.44**	**1.271–1.631**	**<0.001**
Chemotherapy (yes/no)	**2.08**	**1.813–2.386**	**<0.001**	**2.066**	**1.802–2.370**	**<0.001**
Tumour size (cm)						
≤3.3	1					
3.3–7.5	0.874	0.746–1.024	0.096			
>7.5	1.174	0.970–1.421	0.099			
LNR						
≤4.9%	1					
4.9–32.8%	**1.454**	**1.123–1.884**	**0.005**	**1.457**	**1.125–1.888**	**0.004**
>32.8%	**2.318**	**1.708–3.146**	**<0.001**	**2.329**	**1.716–3.160**	**<0.001**
Perineural invasion (no/yes)	**1.307**	**1.136–1.504**	**<0.001**	**1.296**	**1.127–1.491**	**<0.001**

**Fig. 5:**
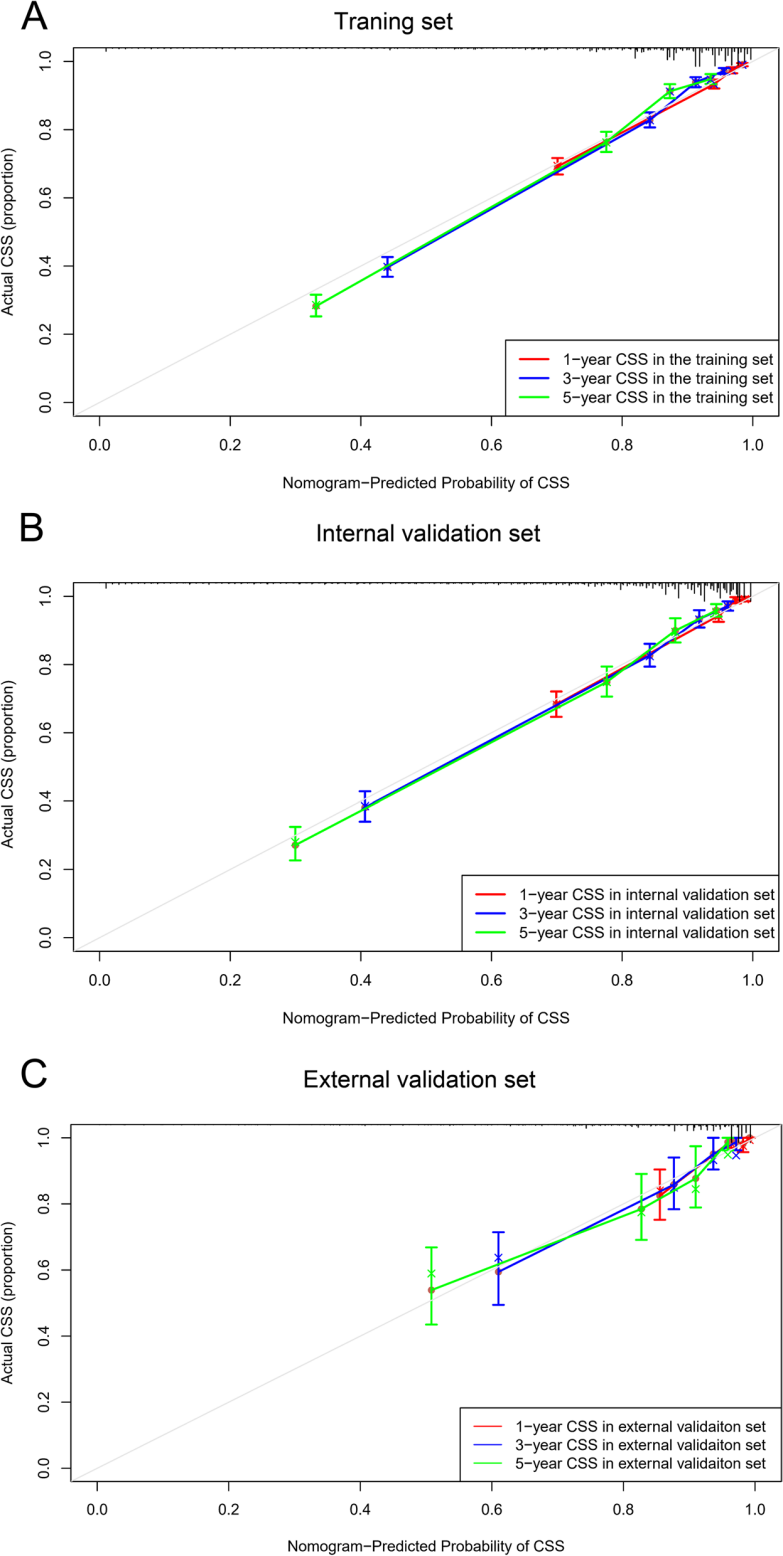
Calibration plots of the nomogram for predicting 1-, 3- and 5-year CSS in the training (**A**), internal validation (**B**) and external validation (**C**) sets. The dashed line represents a perfect match between actual CSS outcome (*Y*-axis) and nomogram prediction (*X*-axis)

**Fig. 6:**
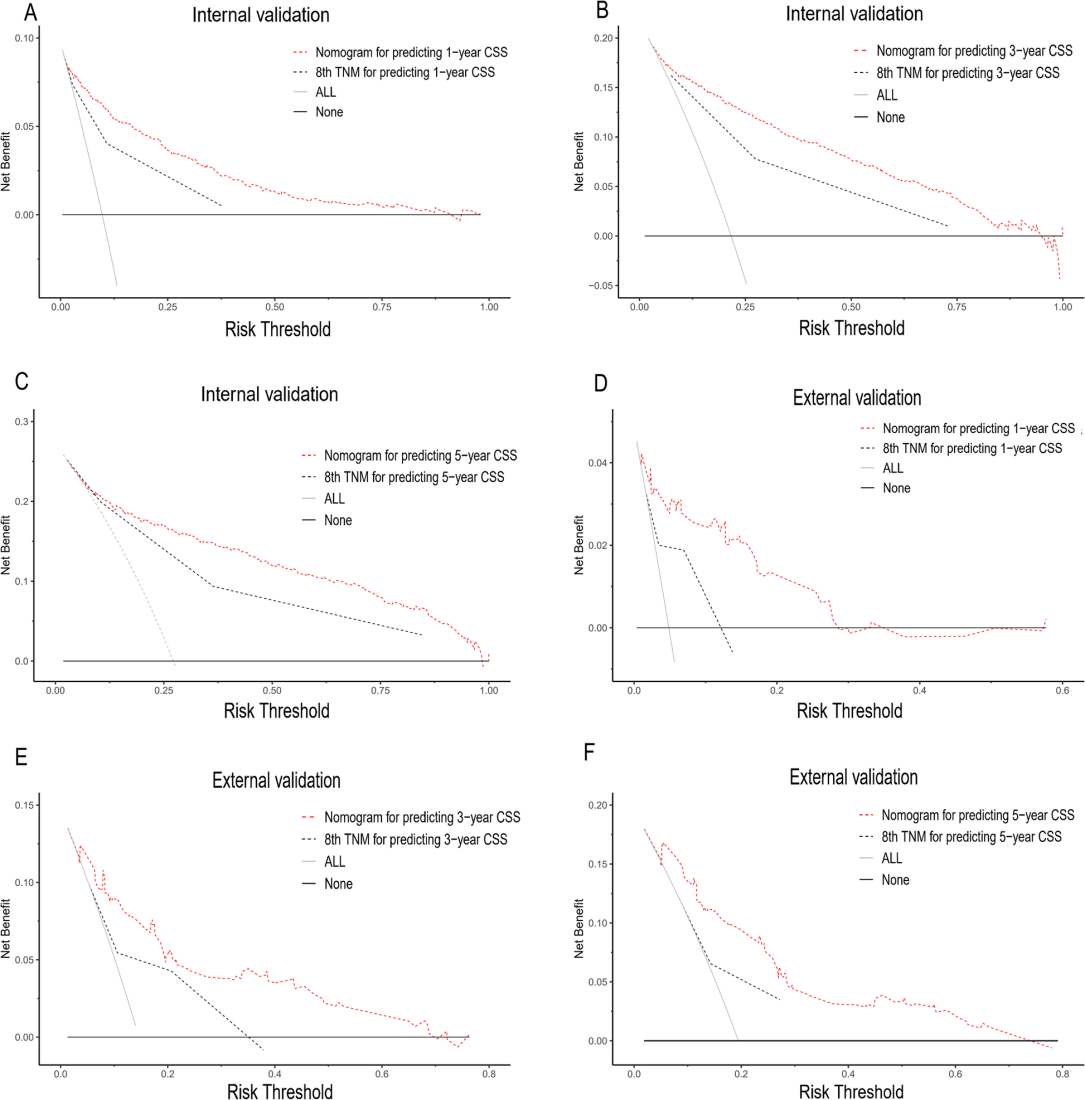
Decision curve analysis of 1-, 3- and 5-year CSS predicting the nomogram and TNM stage system in the internal validation (**A**, **B** and **C**) and external validation sets (**D**, **E** and **F**). The *x*-axis shows the threshold probabilities. The *y*-axis represents net benefit. The grey line indicates the assumption that all patients died from ascending colon adenocarcinoma. The black line indicates the assumption that all patients survived

The calibration curves showed good agreement between the nomogram predictions and the actual proportion in both the training set and the validation set (Fig. 5). The DCA curve, which evaluates models from the perspective of clinical consequence, confirmed the clinical validity of our nomogram for CSS, and our nomogram yielded superior clinical net benefit compared with the AJCC 8th edition TNM staging system (Fig. 6).

## Discussion

Nomograms are more accurate than traditional staging systems in predicting the prognosis of various tumours [16–18]. In this study, a novel nomogram that incorporates haematological biomarkers, clinical features and clinicopathological factors was well developed in predicting CSS for patients with ascending colon adenocarcinoma undergoing surgery and was further verified in the validation set.

The training and internal validation sets were completely randomized from the same respective population (SEER), so the C-index of the two sets did not differ much in this study (0.835 vs. 0.848). Owing to ethnic and geographical differences, the C-index of the external set (0.732) was inferior to the internal validation set but still higher than TNM stage (0.701). Both internal validation and external validation presented higher discrimination and satisfactory calibration and achieved a better and more robust predictive performance than that of the 8th edition of the AJCC TNM staging system.

Patients with right-sided colon cancer exhibit more advanced tumour stages and poorly differentiated tumours and, more commonly, abdominal implantation compared with left-sided colon and rectal cancer [19]. Many earlier studies reported that right-sided colon patients were more likely to be female [19–22], and 4813 female patients were enrolled in our study, accounting for 53.6% of all the patients included in this study. The results of our study support the conclusions described above. The literature has reported that patients older than 60 years make up 65% of the cases of carcinoma of the ascending colon [23]. There was a linear relationship between age and location of colon carcinoma: an older age was more common and more proximal to the tumour [7]. Likewise, our study also demonstrated that older age, with cut-off values of 73 and 85, could act as an independent factor influencing the prognoses of patients with ascending colon cancer after surgery.

Perineural invasion (PNI) is regarded as a reasonable risk factor when incorporating all possible ways of tumour spread, and the prognostic impact of PNI has been demonstrated in many studies, including other cancers [24–26]. The College of American Pathologists has highlighted the importance of PNI and recommended reporting PNI in patients with carcinoma of the colon and rectum since 2009 [27]. In the current study, PNI was also incorporated into the novel nomogram as an independent prognostic factor.

It has been widely recognized that T stage, N stage, M stage and chemotherapy are prognostic factors in patients with colon tumours [28–30]. The level of serum CEA is an important prognostic marker for cancer treatment, recurrence and metastasis [31]. Serum CEA has shown great value for the differential diagnosis of malignant tumours and postoperative prediction of CRC. Konishi et al. [32] showed that elevated CEA is an important predictor of recurrence in colon cancer, which could further affect the CSS of patients. These independent prognostic factors were also included as components of our novel nomogram.

Studies indicate that right-sided tumours exhibit poorer differentiation than left-sided tumours [33, 34]. The degree of differentiation of the tumour is an important index for assessing malignancy risk and disease prognosis. The poorer the tumour differentiation degree is, the more malignant the degree will be, which could reduce the survival rate of patients. Tumour differentiation was regarded as an independent factor in our nomogram. The LNR has been investigated as an important parameter, and the LNR can be used to estimate prognosis and identify high-risk patients [35]. The LNR was an independent value for discriminating survival outcomes and was even more precise than the classic N stage [36]. Our study showed that 4.9% and 52.8% were the cut-off values of LNR, and the range of LNR varied according to the type and site of tumour [37–39], suggesting that the value should be individualized.

Data from various studies are conflicting and contradictory regarding the prognostic significance of tumour size. Some studies have demonstrated that tumour diameter, particularly larger size, was not an influencing factor of prognosis [40, 41], while others identified it as an important influencing factor [42, 43]. The common denominator of these studies above was that subanalysis according to each site was not performed, which might account for the contradiction. A previous study [44] showed that a smaller tumour size (<40 mm) was an independent risk factor for CSS in patients with RCC. Right colon tumours, especially ascending colon tumours, often exhibit an exophytic growth pattern and larger and more advanced stages [45]. Our data derived from SEER indicated that the average tumour size of all patients was 50 mm. Multivariate Cox regression analysis revealed that tumour size was not an independent prognostic factor. As a result, tumour size was not included in the development of the current nomogram for ascending colon tumours exclusively. This conclusion suggested that further subgroup analyses based on the primary tumour location are warranted.

Although our nomogram achieves good prediction, there are several limitations worth noting. First, this is a retrospective study of a large population, so unavoidable confounding factors might limit the validity of this study, and further prospective studies are needed to confirm these findings. Second, the detailed information for chemotherapy and specific values of CEA within the SEER database remain unclear. Third, this model was developed and validated from the SEER database and externally validated in a single centre in China; however, whether the model is applicable to other ethnic or racial groups needs further investigation, and it still requires external validation from other centres before it can be widely utilized in the clinical setting.

## Conclusion

Our study indicates that the novel nomogram could effectively predict the prognosis of patients with ascending colon adenocarcinoma after surgery. The predictive ability of our nomogram is relatively promising, and after more extensive evaluation and broadened analysis from different populations, it may improve the predictive power and assist clinicians in a more precise prediction of prognosis.

## Data Availability

The raw data of this manuscript are available upon reasonable request from the corresponding author.

## Abbreviations

CSS: Cancer-specific survival
SEER: Surveillance, Epidemiology, and End Results
LNR: Lymph node ratio
AJCC: American Joint Committee on Cancer
TNM: Tumour-node-metastasis
CEA: Carcinoembryonic antigen
CRC: Colorectal cancer
ROC: Receiver operating characteristic
AUC: Area under the curve
DCA: Decision curve analysis
CSD: Cancer-associated death
PNI: Perineural invasion
