# Correction: Development and external validation of a novel nomogram for predicting cancer-specific survival in patients with ascending colon adenocarcinoma after surgery: a population-based study

**DOI:** 10.1186/s12957-025-03685-6

**Published:** 2025-02-14

**Authors:** Yi Fan Zhang, Cheng Ma, Xiao Ping Qian

**Affiliations:** 1 Comprehensive Cancer Center, Nanjing Drum Tower Hospital Clinical College of Nanjing Medical University, Nanjing, 210000 China; 2 Department of Radiotherapy, The Xuzhou School of Clinical Medicine of Nanjing Medical University, Xuzhou, 221000 China; 3 Department of Gastrointestinal Surgery, The Xuzhou School of Clinical Medicine of Nanjing Medical University, Xuzhou, 221000 China; 4 Comprehensive Cancer Center, Nanjing Drum Tower Hospital, Medical School of Nanjing University, Clinical Cancer Institute of Nanjing University, Nanjing, 210000 China

**Correction: World J Surg Onc 20, 126 (2022)**

10.1186/s12957-022-02576-4

Following publication of the original article [1], the author reported that the affiliations for the first author should be updated.

From

1.Department of Radiotherapy, The Xuzhou School of Clinical Medicine of Nanjing Medical University, Xuzhou, 221,000, China.

2.Comprehensive Cancer Center, Nanjing Drum Tower Hospital Clinical College of Nanjing Medical University, Nanjing, 210,000, China.

To

1.Comprehensive Cancer Center, Nanjing Drum Tower Hospital Clinical College of Nanjing Medical University, Nanjing, 210,000, China.

2.Department of Radiotherapy, The Xuzhou School of Clinical Medicine of Nanjing Medical University, Xuzhou, 221,000, China.

This has been updated above and original article has been updated.
